# Emerging Targets in Type 2 Diabetes and Diabetic Complications

**DOI:** 10.1002/advs.202100275

**Published:** 2021-07-28

**Authors:** Sevgican Demir, Peter P. Nawroth, Stephan Herzig, Bilgen Ekim Üstünel

**Affiliations:** ^1^ Institute for Diabetes and Cancer (IDC) Helmholtz Center Munich Ingolstädter Landstr. 1 Neuherberg 85764 Germany; ^2^ Joint Heidelberg ‐ IDC Translational Diabetes Program Internal Medicine 1 Heidelberg University Hospital Im Neuenheimer Feld 410 Heidelberg 69120 Germany; ^3^ DZD Deutsches Zentrum für Diabetesforschung Ingolstädter Landstraße 1 Neuherberg 85764 Germany; ^4^ Department of Internal Medicine 1 and Clinical Chemistry Heidelberg University Hospital Im Neuenheimer Feld 410 Heidelberg 69120 Germany

**Keywords:** type 2 diabetes, diabetic complications, insulin resistance, metabolism, signaling pathways

## Abstract

Type 2 diabetes is a metabolic, chronic disorder characterized by insulin resistance and elevated blood glucose levels. Although a large drug portfolio exists to keep the blood glucose levels under control, these medications are not without side effects. More importantly, once diagnosed diabetes is rarely reversible. Dysfunctions in the kidney, retina, cardiovascular system, neurons, and liver represent the common complications of diabetes, which again lack effective therapies that can reverse organ injury. Overall, the molecular mechanisms of how type 2 diabetes develops and leads to irreparable organ damage remain elusive. This review particularly focuses on novel targets that may play role in pathogenesis of type 2 diabetes. Further research on these targets may eventually pave the way to novel therapies for the treatment—or even the prevention—of type 2 diabetes along with its complications.

## Graphical abstract

Type 2 diabetes is a complex metabolic disorder associated with a plethora of complications in peripheral organs. This review article highlights the novel targets that might play role in pathogenesis of type 2 diabetes. Further research on these targets might eventually pave the way to novel therapies for the treatment–or even the prevention–of type 2 diabetes.

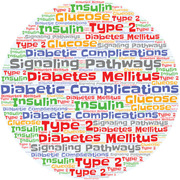

## 1 Introduction

Diabetes mellitus is a chronic, metabolic disorder characterized by abnormally high blood glucose levels known as hyperglycemia. The Greek word diabetes means *to* siphon or to pass through and the Latin word mellitus means sweet, referring to high sugar levels in the urines of patients with diabetes. The earliest mention of diabetes dates back to 1552 BC written on an Egyptian papyrus, making it one of the oldest diseases described in human history. Initial attempts for treating diabetes mainly focused on herbal extracts and dietary interventions. Patients with diabetes had very poor prognosis with very low quality of life and particularly it used to be a death sentence for children. It was not until the discovery of insulin in 1921 by Frederik G. Banting and Charles Best at the University of Toronto, when life‐saving treatments started to take off.^[^ ^1^, ^2^ ^]^ Later in 1923, Banting and John Macleod received the Nobel Prize in Physiology or Medicine for their discovery of insulin. Banting shared his winnings with his assistant Best, Macleod, on the other hand, shared it with James Collip, with whose help insulin was successfully purified.

We can describe diabetes as a disease of insulin insufficiency or impaired insulin action. Mainly, two main types of diabetes exist: type 1 and type 2. Type 1 diabetes develops at early stages of life due to an auto‐immune disorder where the cells of the immune system attack the insulin producing *β* cells of the pancreas. Type 2 diabetes, however, develops later in life, due to systemic dysfunctions in metabolic homeostasis. Genetic background plays a critical role in predisposing individuals to type 2 diabetes, where unhealthy eating habits and sedentary life style act as powerful triggers.^[^ ^3^, ^4^ ^]^ Unlike type 1 diabetes, type 2 diabetes is relatively heterogeneous and very complex, involving too many pathophysiological mechanisms that not only affect pancreas but also the metabolic organs, making effective treatment very challenging.

In 2018, Groop and colleagues stratified patients with type 2 diabetes into five different subgroups based on six variables: age at diagnosis, body‐mass index (BMI), insulin resistance, beta cell function, Hb1Ac levels, and glutamate decarboxylase antibodies. Each cluster represented a specific subset of patients with differing risk for particular diabetic complications, which were: 1) severe autoimmune diabetes; 2) severe insulin‐deficient diabetes; 3) severe insulin‐resistant diabetes; 4) mild obesity‐related diabetes; and 5) mild age‐related diabetes.^[^ ^5^ ^]^ Similar to type 2 diabetes patients, individuals that are not yet diagnosed but are at a high risk of developing it, were also stratified into six different subgroups that could predict the complications such as diabetic kidney disease without rapid progression to overt type 2 diabetes.^[^ ^6^ ^]^ These findings indicate that the pathophysiological variation between individuals already exists before type 2 diabetes develops. These findings by independent groups once again provide the evidence for heterogeneity and complexity of type 2 diabetes most likely due to aberrant regulation of different signaling pathways in different target tissues. For instance, it is possible that in severe autoimmune diabetes, defective immune system is responsible for development of type 2 diabetes, whereas in mild age‐related diabetes, pathways that play role in aging and cell senescence in *β*‐cells might play a role. Dissecting these tissue‐specific signaling pathways and identifying novel targets that contribute to type 2 diabetes will definitely, in the future, improve the current taxonomy of diabetes and contribute to precision medicine.

Although the precise definition of sub‐clusters is still a matter of debate and it may take some time to establish protocols and categorize the patients, such stratification will certainly contribute to identify patients that are at risk for developing type 2 diabetes and diabetic complications, which will lead to personalized diabetes therapies, which unfortunately do not exist yet.

Diabetes is a global endemic. In 2019, 463 million of adults (20–79 years old) were living with diabetes; and again–only in–2019, diabetes caused 4.2 million deaths. The number of patients with diabetes is increasing at a very high rate, estimated to reach 700 million by 2045. Diabetes is not only about high blood glucose levels. Patients with diabetes also suffer from a number of complications, which are sometimes already present when diabetes is diagnosed such as diabetic retinopathy; or they develop later during the course of the disease.^[^ ^7^, ^8^ ^]^ These complications involve dysfunctions in many vital organs all over the body; mainly kidney, cardiovascular system, retina, and the nervous system. Fibrosis of the liver and fibrosis of the lungs as well as cognitive dysfunction are also emerging as novel pathologies that develop secondary to diabetes.

In this review, we will introduce the novel targets/concepts that play role in pathogenesis of type 2 diabetes and the diabetic complications both in the context of peripheral organs and *β*‐cells of the pancreas. We will initially focus on insulin and glucagon signaling pathways which are deregulated in type 2 diabetes. We will discuss insulin resistance in metabolic organs liver, skeletal muscle, and adipose tissue separately due to the tissue specific mechanisms. Then, we will discuss the role of *β*‐cell dysfunction in pathogenesis of type 2 diabetes.

Finally, we will give an overview of the state‐of‐the‐art in our current understanding of diabetic complications in peripheral organs including the kidney, cardiovascular system, retina, nerve, and liver.

For this review article, we particularly focused on publications that emerged after 2016. Due to immense number of articles, we specifically chose the targets that showed compelling in vivo evidence regarding their potential role in development of type 2 diabetes and its late complications, summarized in **Table** **1**.

**Table 1:** List of novel targets with emerging implications in type 2 diabetes

Section described	Target	Effect/Potential role	Reference
Insulin signaling pathway	Amlexanox inhibition of TBK1/IKKe	Alleviates obesity related metabolic dysfunctions	^[^ ^13^ ^]^
	p66Shc	Glucose and lipid homeostasis	^[^ ^18^ ^]^
	Nuclear insulin receptor (IR)	Glucose and lipid metabolism, protein synthesis	^[^ ^19^ ^]^
Insulin resistance in liver	IQGAP1	Induces insulin resistance and glucose intolerance	^[^ ^22^ ^]^
	TSC22D4	Promotes insulin resistance and glucose intolerance	^[^ ^27^ ^]^
	CHOP	Apoptotic cell death due to chronic unfolded protein response	^[^ ^36^ ^]^
	Vitamin D receptor (VDR)	Blunts ER stress and UPR	^[^ ^37^ ^]^
	Them2/PC‐TP	Reduce ER stress and enhances hepatic insulin resistance	^[^ ^38^ ^]^
	Cx43	Plays role in ER stress dissemination to adjacent cells	^[^ ^39^ ^]^
	Differential expression of IRS1 and IRS2	Plays role on distinction of gluconeogenic and lipogenic program	^[^^40^, ^41^^]^
Insulin resistance in skeletal muscle	Glut4 specific motifs	Modulates Glut4 trafficking	^[^ ^44^ ^]^
	Non‐canonical PI3K‐Rac1‐PAK1 signaling	An alternative axis for GSC translocation upon insulin engagement	^[^ ^46^ ^]^
	ApoJ	A novel hepatokine regulating muscle glucose and lipid metabolism	^[^ ^48^ ^]^
	LRP2	Required for insulin‐induced IR internalization	^[^ ^48^ ^]^
	Lkb1	Skeletal muscle protein homeostasis	^[^ ^49^ ^]^
	*β*‐AR agonist 5’HOD	Promotes anabolic functions in muscle	^[^ ^52^ ^]^
	Quercetin	Suppresses muscle atrophy	^[^ ^53^ ^]^
	Myostatin	Suppresses muscle growth	^[^^54^, ^55^, ^56^^]^
Insulin resistance in adipose tissue	CCL2	Macrophage infiltration into adipose tissue insulin resistance	^[^ ^57^ ^]^
	ANT2	Increases adipose tissue hypoxia	^[^ ^64^ ^]^
	LTB4/LTB4R1	Leukocyte infiltration into adipose tissue and cytokine production	^[^ ^66^ ^]^
	miR‐155	Exacerbates insulin resistance	^[^ ^67^ ^]^
	Sphk1	Promotes inflammation in adipose tissue and glucose intolerance	^[^ ^68^ ^]^
	DES1	Causes insulin resistance	^[^ ^69^ ^]^
Glucagon signaling	Klf9	Regulates PGC1alpha	^[^ ^81^ ^]^
	*β*‐arrestin 2	Regulates GcgR	^[^ ^88^ ^]^
	SRI‐37330	Promotes glucose handling in T1D and T2D	^[^ ^89^ ^]^
	GLP‐1R/GcgR	Regulates hyperglycemic effects of glucagon action	^[^ ^91^ ^]^
Role of *β*‐cells in T2D	Inceptor	Inhibits INSR and IGFR1	^[^ ^103^ ^]^
	PLCDX3	Promotes GSIS and insulin content	^[^ ^104^ ^]^
	NGF	Promotes glucose induced insulin secretion in *β*‐cells	^[^ ^110^ ^]^
	TrkA	Promotes insulin granule exocytosis	^[^ ^110^ ^]^
	Tcf7l2	Regulates glucose handling and beta cell function	^[^ ^111^ ^]^
Diabetic complications	Methylglyoxal modifications	Increase upon hyperglycemic flux and impaired detoxification	^[^^122^, ^123^, ^124^, ^125^, ^126^^]^
Diabetic kidney disease	Angiotensin II	Induces ROS production and activates TGF*β*1 signaling	^[^ ^142^ ^]^
	SMPDL3b	Impaires insulin/Akt signaling in podocytes	^[^ ^152^ ^]^
	JAML	Promotes excessive lipid accumulation and renal lipotoxicity	^[^ ^154^ ^]^
	VEGF‐B	Elevates glomerular lipid content and causes insulin resistance	^[^ ^128^ ^]^
	Ketone Bodies	Blunt hyperactivated mTORC1 signaling and attenuate renal damage	^[^ ^161^ ^]^
Cardiovascular complications	QKI‐7	Promotes mRNA degradation of essential genes for EC function	^[^ ^176^ ^]^
	Endothelin B receptor	Increases NO levels to protect against the proatherogenic insults	^[^ ^177^ ^]^
	Sarcolipin	Causes diabetic heart failure	^[^ ^178^ ^]^
	HDAC4	Protects from diabetic heart failure	^[^ ^179^ ^]^
	Exophers	Maintain a healthy heart function	^[^ ^180^ ^]^
Diabetic retinopathy	Sema4d	Biomarker for anti‐VEGF‐1 therapy	^[^ ^183^ ^]^
	Ang1	Promotes TGF*β* and PDGF signaling	^[^ ^184^ ^]^
	Ang2	Promote blood retina barrier permeability	^[^ ^185^ ^]^
	circRNA‐cPWWP2A	Impair miR‐579 function and upregulate Ang1/Occludin/SIRT1 expression	^[^ ^186^ ^]^
	circRNA‐cZNF532	Regulates pericyte function and vascularization	^[^ ^187^ ^]^
	Prostaglandin E2 and its receptor	Induces L1*β* and inflammasome NLRP3‐ASC signaling	^[^ ^190^ ^]^
	Ceramide 6	Impairs JNK function and prevents apoptosis	^[^ ^191^ ^]^
	DHA and EPA	Plays protective role in pathogenesis of diabetic retinopathy	^[^ ^192^ ^]^
	12‐HETE or 15S‐HETE	Exacerbate the progression of diabetic retinopathy	^[^ ^192^ ^]^
	Linagliptin	Shows anti‐angiogenic effects	^[^ ^193^ ^]^
Diabetic neuropathy	Na(v)1.8	Increases hyperalgesia	^[^ ^197^ ^]^
	HCN2	Increases hyperalgesia	^[^ ^198^ ^]^
	CXCL12/CXCR4	Promotes initiation of mechanical allodynia	^[^ ^199^ ^]^
	Notch1 or TLR4	Alleviates mechanical allodynia and thermal hyperalgesia thresholds	^[^ ^204^ ^]^
Liver fibrosis	circRNA‐SCAR	Inhibits mitochondrial ROS output and fibroblast activation	^[^ ^207^ ^]^
	AMPK‐Caspase signaling	Inhibits inflammation and liver damage by controlling apoptosis	^[^ ^208^ ^]^
	TAZ	Promotes the expression of pro‐fibrogenic genes and proliferation	^[^ ^210^ ^]^
Other complications of T2D	RAGE	DNA damage repair pathway and lung fibrosis	^[^^213^, ^214^^]^

## 2 Insulin and Insulin Signaling Pathway

Insulin (literally meaning island in Latin) is a peptide hormone produced and secreted by the *β*‐cells of the pancreas upon elevated blood glucose levels. Insulin acts on metabolic organs such as liver, skeletal muscle, and adipose tissue to promote storage of glucose in the form of glycogen and/or lipids, lowering blood glucose levels. Insulin also crosses the blood‐brain‐barrier regulating key functions in the central nervous system such as food intake, peripheral metabolism, memory, and cognition.^[^ ^9^ ^]^

**Figure 1:**
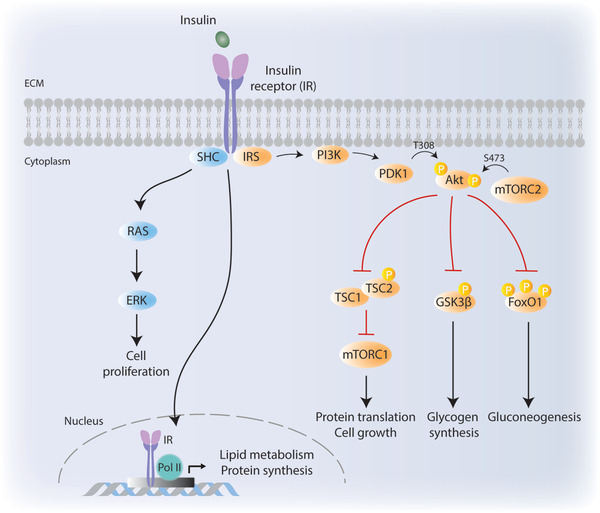
Canonical insulin signaling pathway. Binding of insulin to insulin receptor (IR) triggers phosphorylation of IRS, which in turn phosphorylates PI3K. Activated PI3K recruits PDK1 to the cell membrane. Akt is phosphorylated by PDK1 (on T308) and mTORC2 (on S473). Activated Akt targets a wide range of downstream targets including TSC2, GSK3*β*, and FoxO1 to regulate essential metabolic events. Insulin binding to its receptor also activates SHC adaptor proteins which target RAS and ERK to promote cell proliferation. Activated IR can also translocate to cell nucleus to induce the expression of genes that play role in lipid metabolism and protein synthesis. ECM, extracellular matrix.

When bound by insulin, insulin receptor (IR), a receptor tyrosine kinase, homodimerizes, and autophosphorylates to recruit and phosphorylate its mediator proteins insulin substrate 1 and 2 (IRS1/2) on tyrosine residues (**Figure** **1**).^[^ ^10^ ^]^ IRS1/2 in turn recruits the lipid kinase phosphatidylinositol 3‐kinase (PI3K) to the cell membrane, which phosphorylates the lipid phosphatidylinositol (3,4)‐bisphosphate (PIP_2_) and converts it to phosphatidylinositol (3,4,5)‐trisphosphate (PIP_3_). PIP_3_ in turn recruits 3‐phosphoinositide‐dependent protein kinase 1 (PDK1) and protein kinase B (PKB)/Akt to the cell membrane where PDK1 and mechanistic target of rapamycin complex 2 (mTORC2) phosphorylate Akt on T308 and S473, respectively.^[^ ^10^ ^]^ Double phosphorylation of Akt leads to its full activation, which phosphorylates a wide range of targets including glycogen synthase kinase 3 *β* (GSK3*β*), forkhead box protein O1 (FoxO1), and tuberous sclerosis complex 2 (TSC2) to promote glycogen and lipid synthesis, protein translation, cell growth, and glucose uptake (Figure 1).^[^ ^10^ ^]^ When cells are simulated with insulin, activated Akt phosphorylates TSC2 on several residues to impair the function of TSC complex, leading to mTORC1 activation and subsequent phosphorylation of mTORC1 downstream targets including p70 ribosomal protein S6 kinase 1 (S6K1) and 4E binding protein (4E‐BP1). The non‐canonical I*κ*B kinases IKK*ε* and TANK‐binding kinase 1 (TBK1) directly phosphorylate mTOR within its kinase domain and promote mTORC1 signaling to its downstream targets.^[^ ^11^, ^12^ ^]^ Indeed amlexanox, an inhibitor of IKK*ε*/TBK1 kinases, alleviates obesity related metabolic dysfunctions such as liver steatosis and adipose tissue inflammation while promoting weight loss and insulin sensitivity not only in mice but also in a subset of obese type 2 diabetes patients.^[^ ^13^ ^]^ When bound by insulin, IR also recruits and phosphorylates Shc adaptor proteins p46 and p52, which in turn navigate the insulin signaling toward RAS dependent ERK activation to promote cell proliferation.^[^ ^14^ ^]^ p66^Shc^, another isoform of Shc proteins, on the other hand, plays role in metabolic regulation and energy expenditure in metabolic tissues such as liver, muscle, and brown adipose tissue.^[^ ^15^, ^16^, ^17^ ^]^ Yet whether p66^Shc^ alleviates or exacerbates metabolic disorders remains elusive as almost all of the in vivo studies depend on p66^Shc^ whole body knockouts. It will be critical in the future to create the tissue‐specific p66^Shc^ knockout mouse models to dissect its role in glucose and lipid homeostasis in corresponding organs.^[^ ^18^ ^]^

Very recent findings indicate that IR also translocate to nucleus where it directly engages at transcriptionally active promoters together with DNA polymerase II (Figure 1).^[^ ^19^ ^]^ Target promoters that IR binds to include genes that regulate lipid metabolism and protein synthesis as well disease related genes implicated in diabetes, neurodegeneration, and cancer. Parallel to its role at the cytoplasm, IR localization to nucleus elevates upon insulin stimulation and its nuclear re‐localization is impaired in insulin resistant ob/ob mouse livers.^[^ ^19^ ^]^

## 3 Type 2 Diabetes: At the Crossroads of Insulin Resistance and Glucagon Action

Type 2 diabetes is a very heterogenous and complex disease that develops due to aberrant regulation of many signaling pathways. In this section, we will describe how insulin resistance develops in metabolic organs and what glucagon does in return.

### 3.1 Insulin Resistance in Liver

Under conditions of over nutrition, high blood glucose levels oblige pancreas to produce and secrete more insulin. Constitutive activation of insulin signaling pathway at target tissues due to increased and sustained insulin levels, initiates several negative feedback loops, putting brakes on the initial steps of insulin signaling, contributing to pathological condition known as insulin resistance.

One of the well described negative feedback loops takes place in response to overnutrition‐induced constitutive mTORC1 activation which leads to inhibitory phosphorylations on IRS‐1 by S6K1.^[^ ^20^ ^]^ mTORC1 itself also phosphorylates IRS1 to promote its proteasome‐dependent degradation.^[^ ^21^ ^]^

**Figure 2:**
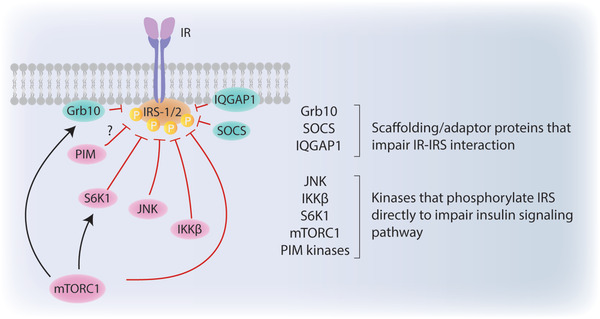
Insulin resistance at IRS‐1/2. Insulin receptor substrate‐1/2 (IRS‐1/2) is a critical target that can be phosphorylated by various kinases to regulate its interaction with insulin receptor (IR). As Grb10, SOCS, or IQGAP1 proteins impair IR‐IRS1/2 interaction; JNK, IKK*β*, S6K1, mTORC1, and PIM kinases phosphorylate IRS‐1/2 to promote its proteasomal degradation.

IRS‐1, indeed, acts as a critical target where independent signaling pathways merge on to establish an insulin resistant state (**Figure** **2**). The effects of S/T phosphorylations on IRS1 function are multifactorial: First, these phosphorylations might impair the IRS1–IR interaction and impair IR induced IRS1 tyrosine phosphorylations. Indeed, hepatocyte specific deletion of IQGAP1 scaffolding protein, which enables IR and IRS‐1 to interact, induces insulin resistance and glucose intolerance in vivo.^[^ ^22^ ^]^ Second, S/T phosphorylations on IRS1 also promote its ubiquitin dependent proteasomal degradation.^[^ ^21^ ^]^ Hyperactivated mTORC1 signaling also contributes to insulin resistance by phosphorylating and stabilizing Grb10 adaptor protein, which impairs IR‐IRS1 interaction.^[^ ^23^, ^24^, ^25^ ^]^ Similarly, suppression of cytokine signaling (SOCS) scaffolding proteins impair the IR‐IRS‐1 interaction and promote degradation of IRS‐1.^[^ ^26^ ^]^

Recently our lab has identified transforming growth factor‐*β* stimulated clone 22 D4 (TSC22D4) as a novel regulator of insulin resistance and hyperglycemia in mouse models of type 2 diabetes. Interestingly hepatic TSC22D4 expression positively correlates with insulin resistance in obese patients and liver specific TSC22D4 knockdown in diabetic mice improves glucose homeostasis and insulin resistance.^[^ ^27^ ^]^

In addition, inflammatory signals initiated by tumor necrosis factor *α* (TNF*α*), interleukin (IL)‐1ß, and IL6 cytokines merge on inhibitor of nuclear factor kappa‐B kinase subunit beta (IKKß) and jun N‐terminal kinase (JNK) signaling pathways to induce inhibitory phosphorylations on IRS‐1.^[^ ^28^, ^29^ ^]^ PIM kinases are also emerging as novel kinases responsible for IRS1 S1101 phosphorylation, yet the implications of these in metabolic regulation still remains elusive.^[^ ^30^ ^]^

Chronic accumulation of unfolded proteins results in inflammatory responses in the cells leading to metabolic diseases such as type 2 diabetes and obesity. Endoplasmic reticulum (ER) is essentially responsible for protein synthesis and protein folding. In response to accumulation of unfolded or misfolded proteins in ER lumen, ER stress leads to unfolded protein response (UPR) to prevent additional injury to the cell.^[^ ^31^, ^32^, ^33^ ^]^ In liver, ER stress results in insulin resistance by impairing regulation of gluconeogenesis and lipogenesis. Each of the UPR proteins has distinct effects on metabolic gene regulation. cAMP‐responsive element binding protein hepatocyte specific, for instance stimulates gluconeogenesis. X‐binding protein 1 (XBP1), on the other hand, suppresses FoxO1 activation hence indirectly promotes sterol regulatory element binding protein 1c (SREBP1c) to activate lipogenesis. Protein kinase RNA‐like endoplasmic reticulum kinase (PERK)–eukaryotic initiation factor 2 (eIf2*α*) branch also activates SREBP1c, and additionally promotes activating transcription factor 4 (ATF4), which in turn stimulates hepatic lipogenesis.^[^ ^34^ ^]^ Fibroblast growth factor 21 (Fgf21) expression is also upregulated upon ER stress via PERK‐eiF2*α*‐ATF4 branch of UPR.^[^ ^33^ ^]^ Fgf21 counteracts ER stress; and by inhibiting lipogenic program, it stimulates glucose uptake in the cells and alleviates hyperglycemia. Chronic UPR may also result in apoptotic cell death via upregulation of C/EBP homologous protein (CHOP) which is regulated by ATF4 in liver.^[^ ^35^ ^]^ CHOP reduces B‐cell lymphoma 2 (Bcl2) anti‐apoptotic mitochondrial protein expression leading to cytochrome c release and caspase‐3 activation.^[^ ^36^ ^]^

Very recent findings have shown that vitamin D receptor (VDR) blunts ER stress and UPR in the liver. VDR deficiency in VDR KO heterozygous mice not only increased UPR action and induced apoptosis but also promoted activation of pro‐inflammatory cytokines such as Interleukin (IL)‐1*β*, IL‐6, and TNF*α*.^[^ ^37^ ^]^

Elevated amount of saturated free fatty acids (SFA) in the ER membrane promotes ER stress, insulin resistance, and eventually excessive hepatic gluconeogenesis. Thioesterase superfamily member 2 (Them2) is a mitochondria‐associated long‐chain fatty acyl‐CoA and it forms a complex with phosphatidylcholine transfer protein (PC‐TP) to promote *β*‐acid oxidation upon acute ER stress. Them2/PC‐TP complex regulates conversion of SFAs to saturated phospholipids to reduce ER membrane fluidity and ER stress. Them2/PC‐TP complex also enhances Ca^2+^ flux into cytosol which leads to hepatic insulin resistance and gluconeogenesis.^[^ ^38^ ^]^

Cell‐cell communication is critical for maintaining systemic metabolism of cells. Gap junctions (GJ) are essential channels that maintain cell‐cell communication by allowing ions, signaling molecules, and metabolites to enter the adjacent cells. GJ are consisted of connexons, which are formed by six connexin (Cx) subunits. Very recently, connexin 43 (Cx43) emerged as one of the key regulators of ER‐stress induced cell‐cell coupling in hepatocytes in response to obesity. Chronic ER stress promotes expression of Cx43 and, therefore, Cx43‐mediated intercellular trafficking disseminates ER stress toward adjacent cells (“bystander cells”). Since hepatic ER stress and dysfunction play role in regulating stress signals associated to insulin resistance and diabetes, systemic glucose homeostasis become disrupted also in the bystander cells. Indeed, liver specific deletion of Cx43 protects mice from diet induced‐ER stress, insulin resistance, and hepatosteatosis.^[^ ^39^ ^]^

### 3.2 Selective Insulin Resistance

In healthy individuals, fasting increases glucagon secretion from pancreas, which activates gluconeogenic program in metabolic organs, mainly liver and kidney. Gluconeogenesis is the process of de novo glucose synthesis from non‐carbohydrate precursors, including amino acids, pyruvate, lactate, glycerol as well as the intermediates of the Krebs cycle. Sustained gluconeogenesis represents one of the hallmarks of insulin resistance and type 2 diabetes in which liver keeps maintaining gluconeogenic activity despite high glucose levels in the blood, exacerbating hyperglycemic state.

**Figure 3:**
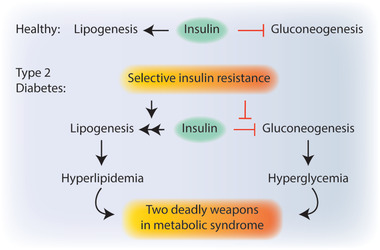
Selective insulin resistance. In healthy individuals, insulin promotes lipogenesis while suppressing hepatic gluconeogenesis to lower the blood glucose levels. In type 2 diabetes, distorted insulin action promotes lipogenesis yet fails to inhibit gluconeogenesis. This phenomenon is known as selective insulin resistance.

In healthy individuals, to lower blood glucose levels, insulin suppresses gluconeogenesis while promoting lipogenesis. In type 2 diabetes, however, insulin action fails to suppress gluconeogenesis, yet it keeps activating lipogenesis, pairing two deadly weapons of metabolic syndrome: “hyperglycemia” and “hyperlipidemia”. This pathogenic paradox, known as selective insulin resistance, represents one of the key questions in metabolic syndrome (**Figure** **3**). Recent studies indicate that differential expression of IRS1 and IRS2 in periportal (PP) and perivenous (PV) zones of the liver creates this distinction between gluconeogenic and lipogenic program. IRS2 localizes in PP and PV whereas IRS1 localizes mainly in PV area, which is responsible for lipogenesis. While hyperinsulinemia‐via negative feedback loops‐leads to a decrease in IRS2 expression in PP and PV zones and relieve the inhibition of gluconeogenesis, it fails to downregulate IRS1 expression in the PV zone, where lipogenic processes still take place.^[^ ^40^ ^]^ Indeed, IRS2 expression is epigenetically repressed in the livers of obese humans with type 2 diabetes.^[^ ^41^ ^]^ Selective insulin resistance is an emerging topic in metabolic syndrome. Understanding exact molecular mechanisms underlying this pathological paradox will be of great benefit in search for novel treatments for metabolic diseases.

### 3.3 Insulin Resistance in Skeletal Muscle

In addition to liver, skeletal muscle also plays a vital role in reducing blood glucose levels by promoting its uptake upon insulin stimulation.^[^ ^42^ ^]^ In skeletal muscle, glucose transporter 4 (GLUT4) is identified as the most abundant glucose transporter isoform. Although a small portion of GLUT4 can be found on the cellular membrane, around 80% of GLUT4 is located in GLUT4 storage vesicles (GSV). In the presence of insulin or upon exercise, GLUT4 translocates from GSVs to muscle cell surface to promote glucose uptake.^[^42^,^ ^43^ ^]^ Upon glucose uptake, skeletal muscle cells either direct the glucose to glycolysis or use it for glycogen synthesis depending on their metabolic needs.

GLUT4 contains specific motifs in the amino cytoplasmic domain (FQQI) and in carboxyl cytoplasmic domain (LL and TELEY) which modulate GLUT4 trafficking from GSVs. Although the roles of certain proteins (GGA, retromer, AP1, etc.) that are interacting with these specific motifs in this trafficking are well known, there are still gaps to complete for GLUT4 translocation machinery including GSV regulations.^[^ ^44^ ^]^

In type 2 diabetes, reduction in insulin's ability to stimulate glucose uptake from peripheral tissues occurs due to the disruption of GLUT4 translocation to the cell surface.^[^ ^45^ ^]^ Muscle GLUT4 emerges as a specific target upon insulin action because exercise‐modulated GLUT4 translocation remains unchanged in type 2 diabetes.^[^ ^44^ ^]^ Since exercise—induced glucose uptake remains preserved in insulin resistant—skeletal muscle, exercise is suggested as a key therapy against metabolic diseases such as type 2 diabetes. Although PI3K‐Akt signaling axis is one of the major pathways activated upon insulin engagement, defects in Akt phosphorylation on both phosphorylation sites (S‐473 and Thr‐308) showed only minor effects toward phosphorylation of Akt downstream targets.^[^ ^44^ ^]^ Other than the canonical PI3K‐Akt‐Rab axis, non‐canonical PI3K‐Ras‐related C3 botulinum toxin substrate 1 (Rac1) – p21‐activated kinase (Pak1) actin remodeling pathway emerges as an alternative axis for GSV translocation upon insulin engagement.^[^ ^46^ ^]^

Accumulation of plasma free fatty acid also causes insulin resistance in skeletal muscle.^[^ ^47^ ^]^ Palmitic acid and stearic acid are some examples of saturated long chain fatty acids (FAs) causing mitochondrial dysfunction and insulin resistance. In mitochondrial dysfunction, not only mitochondrial density decreases in insulin resistant people but also rate of ATP synthesis and oxygen consumption decrease. Elevated reactive oxygen species (ROS) levels resulting from accumulation of FA‐derived metabolites (i.e., diacylglycerol and ceramides), impair mitochondrial biogenesis and activate stress kinases impairing glucose uptake and insulin tolerance.^[^ ^47^ ^]^

Hepatokines, liver derived hormones, are essential for liver‐muscle trafficking; and Apolipoprotein J (ApoJ) emerges as a novel hepatokine targeting muscle glucose metabolism and insulin sensitivity via low density lipoprotein‐related protein 2 (LRP2).^[^ ^48^ ^]^ LRP2 is required for insulin‐induced insulin receptor internalization in skeletal muscle. Muscle specific LRP2 deficiency or hepatic specific ApoJ deficiency promotes glucose intolerance and insulin resistance. ApoJ KO mice have defective insulin signaling with reduced phosphorylation on its canonical targets such as insulin receptor, IRS1/2, Akt and Akt substrate of 160 kDa (AS160) in skeletal muscle. FGF21 and selenoprotein B are other examples of hepatokines directly affecting glucose and lipid metabolism in liver, muscle, and adipose tissue. LRP2 also binds to selenoprotein B and promotes its uptake in kidney.^[^ ^48^ ^]^

Liver kinase B1 (*Lkb1*) suppresses amino acid induced gluconeogenesis in the liver. Hepatocyte specific *Lkb1* deletion showed increased levels of hepatic amino acid catabolism, inducing gluconeogenesis. Although *Lkb1* deficiency increased levels of amino acids in liver, it decreased the levels of amino acids in plasma. This metabolic impairment disrupts protein homeostasis in skeletal muscle and contributes to metabolic disorders such as cachexia and sarcopenia.^[^ ^49^ ^]^

Cachexia is a metabolic syndrome that involves extreme body weight loss and muscle wasting. Usually cachexia emerges as a complication of certain diseases such as cancer, AIDS, or chronic kidney disease.^[^ ^50^ ^]^ In very rare cases, cachexia also represents itself as a complication of diabetes also known as diabetic neuropathic cachexia (DNC). The underlying molecular mechanisms that lead to DNC remain elusive. Unlike DNC, sarcopenia is more prevalent among patients with type 2 diabetes. Sarcopenia involves age‐related loss in muscle mass and function due to impaired protein metabolism, mitochondrial dysfunction, and cell death, causing inflammation and impairing skeletal muscle's ability to uptake glucose. Hence, sarcopenia has a bidirectional relationship with type 2 diabetes, that is, while it promotes pathogenesis of type 2 diabetes it might as well emerge due to insulin resistance, oxidative stress, and vascular complications.^[^ ^51^ ^]^

One of the mechanisms that enhances muscle hypertrophy and stimulate skeletal muscle metabolism is via activation of the *β*‐adrenergic receptor (*β*‐AR) signaling pathway and cAMP production. The use of *β*‐AR agonists such as formoterol, however, has been challenging due to its extensive burden in cardiovascular system. Nevertheless, very recently a novel *β*‐AR agonist called 5‐hydroxybenzothiazolone‐derived (5’HOD) has been described which showed superior selectivity for muscle tissue and promoted anabolic functions in the muscle without inducing any side effects in the cardiovascular parameters.^[^ ^52^ ^]^

The increased levels of pro‐inflammatory cytokines such as IL‐6, monocyte chemoattractant protein 1 (MCP‐1), and TNF*α* contributes to sarcopenia by inducing muscle wasting. Recent findings show that quercetin, a flavonoid with anti‐oxidant and anti‐inflammatory features, successfully counteracts the muscle atrophy induced by TNF*α*. Quercetin suppresses the expression of atrophic factors MAFbx/atrogin‐1 and Muscle RING Finger‐1 (MuRF1) while promoting the function of heme oxygenase ‐1 (HO‐1) and Nrf‐2.^[^ ^53^ ^]^

Ectopic accumulation of lipids in the skeletal muscle also induces inflammation, oxidative stress, and lipotoxicity, impairing insulin‐dependent glucose uptake and mitochondrial function, overall contributing to insulin resistance. A critical upstream regulator of these cellular functions is a protein called myostatin, which is upregulated under conditions of metabolic syndrome. Myostatin impairs Akt and AMPK function to downregulate muscle growth. Inhibition of myostatin function in mice increased muscle mass and improved insulin sensitivity.^[^ ^54^, ^55^, ^56^ ^]^

### 3.4 Insulin Resistance in Adipose Tissue

Adipose tissue is spread all over the body with different types and unique features regulating metabolic activities. While brown adipose tissue (BAT) maintains lipogenic program upon changing thermogenic activities, lipids are stored mainly in white adipose tissue (WAT) which has two subtypes: the visceral WAT (vWAT) and the subcutaneous WAT (scWAT). In metabolic disorders, vWAT secretes IL‐6 and plasminogen‐activator inhibitor (PAI‐1) into portal system. On the other hand, scWAT expresses leptin and adiponectin and secrete the adipokines into systemic circulation for maintaining metabolic homeostasis.^[^ ^57^ ^]^

The trafficking between adipocyte‐hepatocyte differs in fasted and fed state of the cells. In the fasted state, adipocytes produce glycerol and release nonesterified fatty acids (NEFA) into circulation. In hepatocytes, while glycerol promotes gluconeogenesis directly; NEFAs are processed through *β*‐oxidation to produce acetyl CoA, which in turn activates pyruvate carboxylase to stimulate gluconeogenesis.^[^ ^43^ ^]^ On the other hand, upon insulin binding (fed state), insulin‐IR‐Akt axis activates mTORC1 which stimulates SREBP1c in liver inducing de novo lipogenesis (DNL).^[^ ^57^ ^]^ Liver packs triglycerides into very low‐density lipoproteins (VLDL) and secretes them into circulation to be taken up by skeletal muscle and adipocytes via CD36 and lipoprotein lipase (LPL) action.^[^ ^43^ ^]^ Consequently, insulin stimulation suppresses NEFA and glycerol release from WAT into the circulation.^[^ ^43^ ^]^

Additional to overnutrition, aging also promotes senescence in type 2 diabetes associated organs, and type 2 diabetes patients experience relatively rapid aging.^[^ ^58^ ^]^ Secreted pro‐inflammatory cytokines and changing metabolites upon aging result in low‐grade inflammation that manifests itself with hyperglycemia, dyslipidemia, and other metabolic problems.^[^ ^59^ ^]^ Therefore, aging and type 2 diabetes share particular characteristics in expressing high levels of pro‐inflammatory cytokines; for example, IL‐6. Senescence‐associated secretory phenotype (SASP) which is shared in both type 2 diabetes and aging is particularly related to oxidative and ER stress. Together with the state of low‐grade inflammation, the senescent cells eventually become both the cause and the consequence of systemic changes associated in diabetes. Interestingly, leukocyte telomere length (LTL) which is a marker of senescence has been proposed to be used as a marker for type 2 diabetes since some diabetic complications are associated with telomere length.^[^ ^58^ ^]^

As in skeletal muscle, mitochondrial dysfunction occurs in adipocytes leading to ER stress, hypoxia, and fibrosis. Because of metabolic imbalance in adipocytes, various cytokines (e.g., IL‐1, IL‐12) and chemokines (IL‐8) attract immune cells to the peripheral tissues. Synthesized proinflammatory mediators (e.g., TNF*α* and IL‐6) disrupt tissue functions and cause metaflammation, which is a state of chronic and low‐grade inflammation. Excessive nutrient consumption causes metaflammation since the cytokine expression and immune cell infiltration accumulates over time.^[^ ^60^, ^61^, ^62^ ^]^

One of the earlier events that may lead to inflammation is the hypoxic conditions that emerge due to the enlargement of adipose tissue and adipocyte size.^[^ ^63^ ^]^ One of the targets that promote adipose tissue hypoxia is adenine nucleotide translocase 2 (ANT2) which increases adipose tissue oxygen demand. Interestingly, adipocyte specific ANT2 KO mice not only had lower levels of adipocyte hypoxia but also showed improved glucose metabolism and insulin sensitivity.^[^ ^64^, ^65^ ^]^

TNF*α*, which plays role in metabolic alterations in cancer, cachexia, and dyslipidemia, emerges as one of the main mediators that have negative correlations on insulin resistance. TNF*α* neutralization in fat tissue improves insulin sensitivity and glucose handling in obese and diabetic mouse models.^[^ ^62^ ^]^

Infiltration of immune cells, such as macrophages into adipose tissue is one of the characteristics of metaflammation. CC‐motif chemokine ligand 2 (CCL2) is a chemokine expressed in adipocytes and it promotes macrophage infiltration into adipose tissue in obesity‐induced insulin resistance. Leptin also contributes to macrophage infiltration by increasing circulation of proinflammatory mediators upon food intake. Additionally, leptin acts as insulin sensitizer in liver and skeletal muscle and regulates *β* cell activity in pancreas.^[^ ^57^ ^]^

Not only macrophages but also B2 lymphocytes are enriched in obese adipose tissue. B2 cell deficient mice are protected against diet induced insulin resistance. Very interestingly, adoptive transfer of B2 cells from high fat diet (HFD) fed mice to the B‐cell deficient null mice rendered the latter to insulin resistance. B2 cell recruitment to the adipose tissue and its activation was mediated by chemokine leukotriene B4 (LTB4), which binds to LTB4 Receptor 1 on B cells. LTB4/LTB4R1 engagement promotes further leukocyte infiltration into adipose tissue and promotes cytokine production.^[^ ^66^ ^]^

The macrophages that reside in adipose tissue of obese mice secrete miRNA‐containing exosomes, which induce insulin resistance and glucose intolerance when administered to lean mice. Conversely, transfer of these vesicles from lean mice to obese mice improved insulin resistance. One of miRNAs overexpressed in macrophages of obese mice is miR‐155, which targets PPAR*γ* and knockout of miR‐155 in mice improves both glucose handling and insulin sensitivity.^[^ ^67^ ^]^

Sphingosine kinase 1 (Sphk1) regulates sphingolipid metabolism which is essential for cell recognition, stress responses, inflammation, and apoptosis. Sphk1 deficiency decreases inflammation in adipose tissue and protects obese mice from diabetes. Additionally, Sphk1 promotes glucose sensitivity and promotes *β*‐cell survival in diet‐induced obese mice.^[^ ^68^ ^]^ Ceramides are also sphingolipids that excessively accumulate in the adipose tissue due to obesity, impair glucose uptake and exacerbate insulin resistance. The enzyme dihydroceramide desaturase 1 (DES1) plays a role in ceramide synthesis by introducing a conserved double bond into molecules. Interestingly, both whole body and tissue specific (liver and/or adipose tissue) DES1 deficiency improves insulin resistance in mice, suggesting DES1 as a novel target against imbalanced glucose handling and metabolic disorders.^[^ ^69^ ^]^

## 4 Glucagon Signaling

**Figure 4:**
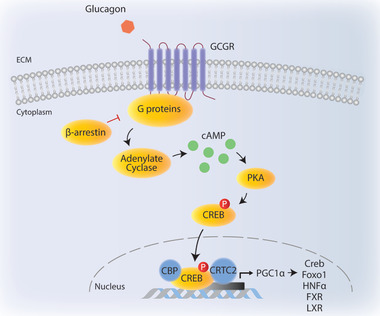
Glucagon signaling. Upon glucagon binding, GCGR activates adenylate cyclase that increases cAMP levels in the cytoplasm. cAMP activates PKA which phosphorylates CREB and leads to its translocation to nucleus. CREB forms a complex with CBP and CRTC2 to regulate gluconeogenic gene expression and fatty acid oxidation via targets such as PGC1*α*, FoxO1, hepatic HNF4*α*, FXR, and LXR. ECM: Extracellular matrix.

When Banting and Best discovered insulin, they also noted that the pancreatic extracts contained hyperglycemic properties as well. In 1922, Kimball and Murlin successfully isolated the fraction that had only hyperglycemic effect and named it glucagon.^[^ ^70^ ^]^ In 1948, Sutherland and de Duve showed that, it is the alpha cells of the pancreatic islets that produce glucagon.^[^ ^70^ ^]^ Although skeletal muscle, heart, kidney, stomach, and small intestine are among the organs that express the glucagon receptor, glucagon exerts its metabolic effects mainly by liver. Glucagon receptor is a seven transmembrane receptor that belongs to G‐protein coupled receptor family. Binding of glucagon to hepatic glucagon receptor activates adenylate cyclase (AC) 5 and AC 6 increasing cellular cAMP levels, which act as a second messenger to activate protein kinase A (PKA) signaling (**Figure** **4**).^[^ ^71^, ^72^ ^]^ cAMP action in liver is very critical during fasting state to ensure glucose‐dependent tissues such as brain and red blood cells have sufficient glucose supply provided by liver.

One of the well‐characterized substrates of PKA is cAMP responsive element binding protein (CREB). CREB acts a transcription factor (binding to promoter regions of genes) that plays key role in gluconeogenesis such as pyruvate carboxylase (PC), phosphoenolpyruvat‐carboxykinase (PEPCK), glucose‐6‐phosphatase (G6Pase), and peroxisome proliferator‐activated receptor‐gamma coactivator‐1alpha (PGC1*α*) (Figure 4).^[^ ^73^, ^74^, ^75^, ^76^, ^77^, ^78^, ^79^, ^80^ ^]^ Recently, Krüppel like factor 9 (Klf9) was identified as a novel upstream regulator of PGC1*α*. Glucocorticoids (i.e., dexamethasone) and fasting upregulates Klf9 gene expression and Klf9 itself binds to the promoter region of PGC1*α* and acts a transcriptional activator. Interestingly liver specific Klf9 deficiency alleviates dexamethasone‐induced hyperglycemia, potentially revealing one of the mechanisms explaining how glucocorticoids might promote diabetes.^[^ ^81^ ^]^

### 4.1 Glucagon Signaling in Diabetes

Unlike in healthy individuals where glucagon levels elevate under conditions of hypoglycemia, some patients with diabetes present increased blood glucagon levels despite hyperglycemia. Persistent exposure to glucagon creates an excessive burden on liver due to ongoing gluconeogenesis and glycogenolysis, which in turn exacerbates hyperglycemia and eventually creating a vicious cycle, contributing to pathological condition known as insulin resistance.

Indeed, approaches antagonizing glucagon signaling as well as studies in glucagon receptor knockout mice lead to promising results in which blood glucose levels decreased while glucose tolerance and insulin sensitivity improved.^[^ ^82^, ^83^, ^84^, ^85^ ^]^ For instance, inhibition of glucagon receptor (GCGR) via overexpression of *β*‐arrestin 2 alleviated metabolic defects in HFD fed mice.^[^ ^86^ ^]^ *β*‐arrestins bind to GCGRs when GCGRs undergo multiple phosphorylation events by glucagon receptor kinases (GRKs) and inhibition of GCGR signaling via *β*‐arrestins involve at least two mechanisms. *β*‐arrestins can either impair the interaction between GCGR and G proteins or promote the internalization of GCGRs via clathrin‐mediated endocytosis leading to a desensitization mode (Figure 4).^[^ ^87^ ^]^ The barcode hypothesis of GCGR refers to the long‐standing idea that specific phosphorylation events on GCGR direct the interaction with corresponding *β*‐arrestins, induce different conformational changes on *β*‐arrestins and dictate which signaling molecules they will recruit to initiate corresponding signaling pathways. Recent studies with atomic‐level simulations and site‐directed spectroscopy showed that the barcode hypothesis might indeed be a valid one and point out that it is not the number of phosphorylation events per se but the position of phosphorylated residues that act as barcodes.^[^ ^88^ ^]^

Although anti‐glucagon approaches in several independent labs alleviated diabetic symptoms in mouse models, developing therapies that target glucagon has been challenging due to its lipogenic potential. Very recently, a high throughput screening of 300.000 compounds led to the discovery of SRI‐37330, an orally bioavailable small molecule. SRI‐37330 treatment ameliorated diabetes both in type 1 and type 2 diabetes mouse models. SRI‐37330 impaired thioredoxin‐interacting protein (TXNIP) function in pancreas, which in turn impaired glucagon secretion from the alpha cells, contributed to lower blood glucose levels.^[^ ^89^ ^]^ Unlike glucagon receptor antagonists, SRI‐37330 did not have any lipogenic effect.^[^ ^90^ ^]^ In fact, if anything, SRI‐37330 reversed hepatic fat accumulation, facilitating its potential use in treatment of type 2 diabetes and fatty liver disease.

Interestingly, glucagon shares the same precursor molecule, which is proglucagon, with glucagon like peptides 1 and 2 (GLP1 and GLP2). Yet, due to tissue specific posttranslational modifications, alpha cells of the pancreas secrete glucagon, whereas L cells of the intestine secrete GLP1 and GLP2. GLP1 represents one of the most characterized incretin hormones, which is secreted postprandially and acts both on central nervous system and peripheral tissues to induce satiety, reduce food intake, and promote insulin secretion from pancreas. Several GLP‐1 receptor (GLP‐1R) agonists are prescribed to patients with obesity and type 2 diabetes. Yet, like any other medication, GLP‐1R agonists are also not without side effects such as nausea, preventing patients from receiving it at higher doses. Combined therapies in the form of rationally designed unimolecular GLP‐1 and GCGR agonism, on the other hand, have a much greater efficacy in reducing body weight and Hb1Ac levels compared to GLP‐1R agonists only. Co‐agonism of GLP1 and glucagon receptors proves to be sufficient to buffer the hyperglycemic effects of glucagon action. Several unimolecular GLP‐1R/ GCGR agonists are currently tested in phases 1 and 2 clinical studies with promising outcomes.^[^ ^91^ ^]^

In addition to GLP1 and GLP2 hormones, the intestine also contributes to metabolic homeostasis via the microbiota it houses. The gut microbiota is not only an important modulator of gut permeability, but also a critical regulator of glucose and lipid metabolism, with potential implications in pathogenesis of type 2 diabetes and its late complications.^[^ ^92^, ^93^, ^94^ ^]^ Many cause‐effect relationships regarding microbiome's potential role type 2 diabetes are derived from rodent models. Clinical studies in humans, also show that there is a clear correlation between different aspects of gut microbiome and metabolic health. Yet, further studies are needed to address whether alterations in gut microbiome in humans is a cause of type 2 diabetes or is an outcome of it.^[^ ^93^, ^95^, ^96^, ^97^, ^98^ ^]^

## 5 Role of *β*‐cells in Type 2 Diabetes

**Figure 5:**
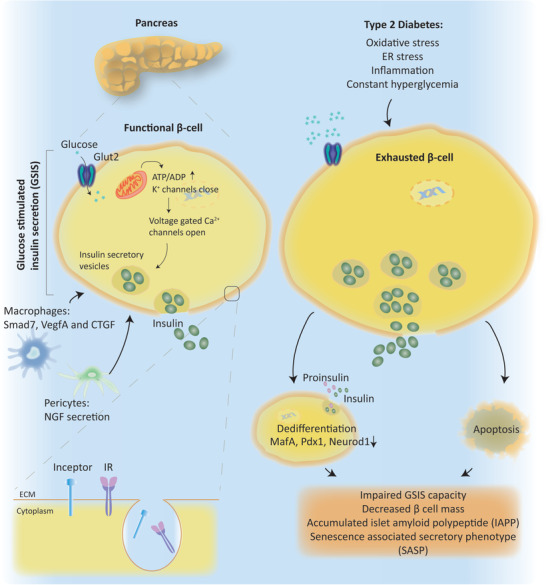
Role of *β*‐cells in type 2 Diabetes. Β‐cells, located in Langerhans islet of pancreas, maintain islet function by regulating insulin release upon glucose stimulation. Glucose stimulated insulin secretion (GSIS), *β*‐cell mass and function are also promoted by different transcription factors regulated via pancreatic macrophages and pericytes. Inceptor, insulin inhibitory receptor, promotes insulin receptor (IR) internalization via clathrin‐mediated endocytosis. Exhausted *β*‐cells in type 2 diabetes increase their number and size to secrete more insulin to blood stream. Challenged *β* cells can either dedifferentiate or undergo apoptosis. Dysfunctional *β* cells cause cytotoxic effects exacerbating type 2 diabetes symptoms.

*β*‐cells, located in Langerhans islet of pancreas, are connected to each other by gap junctions and surrounded by other hormone secreting cells such as *α* (alpha) and *δ* (delta) cells. Thanks to the vascularized structure of the islets, pancreas can maintain islet function by regulating trafficking of secreted growth factors and rapid release of insulin to bloodstream when *β*‐cells sense nutrients. Having appropriate number of functional insulin secreting *β*‐cells (known as *β*‐cell mass) is one of the essential components of insulin secretion. Insulin is secreted via vesicles (insulin secretory granules) and insulin secretion is tightly mediated by regulatory signals. *β*‐cells can sense key regulators such as free fatty acids, amino acids, and hormones such as GLP1 and glucose dependent insulinotropic polypeptide, and most importantly circulating glucose concentration. Glut2 is a transmembrane protein that is abundantly located on *β*‐cell surface and senses the circulating blood glucose levels. Glut2 dependent glucose uptake leads to closure of ATP‐sensitive potassium channels on the membrane (K_ATP_ channels), and opens voltage‐gated calcium channels in return, which leads to secretion of insulin via granules (**Figure** **5**).^[^ ^99^ ^]^

In type 1 diabetes, *β*‐cells are destroyed by autoimmune mechanism leading to apoptosis and causing insulin deficiency. Hence, type 1 diabetes patients need lifelong external insulin treatment. In type 2 diabetes, however, initially *β*‐cells are functional and can still secrete insulin upon high blood glucose concentrations. As the metabolic tissues develop insulin resistance over time, *β*‐cells increase their number and size to secrete more insulin, which puts excessive burden on *β*‐cell function. Over time, *β*‐cells become exhausted, lose their function, stop proliferating which overall decreases *β*‐cell mass.^[^ ^100^ ^]^ Constant hyperglycemic state also causes extra burden on *β*‐cells due to glucotoxicity, which exacerbates *β*‐cell malfunctioning.^[^ ^101^ ^]^

In type 2 diabetes, pancreatic islets have around 60% decrease in *β*‐cell mass and present impaired glucose stimulated insulin secretion (GSIS) capacity. Diabetic islets also contain increased levels of islet amyloid polypeptide (IAPP) compared to non‐diabetic pancreatic islets. Accumulated IAPP has cytotoxic effects and exacerbates *β*‐cell failure by inducing pro‐inflammatory cytokines such as IL‐1*β*, TNF*α*, and IL‐6.^[^ ^101^, ^102^ ^]^

Insulin and insulin growth factor 1 (IGF1) signaling also play a critical role in function and proliferation of *β*‐cells. Recently, scientists discovered an inhibitor of insulin receptor (IR) and IGF1 receptor (IGFR), which they named insulin inhibitory receptor, that is, “inceptor.” Similar to IR and IGF1R, inceptor is also a transmembrane protein located at the cell membrane. Inceptor interacts with IR and IGF1R and promotes endocytosis‐mediated internalization of these receptors leading to their desensitization. Indeed, *β*‐cell specific inceptor knockout mice showed increased IR/IGF1R activation in pancreas, promoted *β*‐cell proliferation and improved glucose homeostasis.**^[^** ^103^ **^]^**

PLCXD3, a member of the phosphoinositide‐specific phospholipases (PI‐PLC) family, also emerges as a novel regulator of genes that play role in insulin signaling pathway. Experiments performed in INS‐1 cells showed that PLCXD3 depletion reduces GSIS and insulin content, and downregulates the expression of genes that play role in insulin synthesis and insulin signaling such as insulin, neuronal differentiation 1 (NEUROD1), GLUT2, glucokinase (GCK), IR, IRS2, and AKT. Indeed, human diabetic islets have reduced PLCXD3 expression, which correlates positively with insulin and GLP1R expression and negatively with donors’ BMI index and HbA1c levels.**^[^** ^104^ **^]^**

Under stress conditions, mature *β*‐cells also lose their differentiated phenotype and dedifferentiate to a precursor‐like state which leads to loss of functional *β*‐cell mass in type 2 diabetes. Additionally, oxidative stress, ER stresses, inflammation, and hypoxic environment stimulates dedifferentiation of *β*‐cells. Factors that lead to dedifferentiation include loss of key *β*‐cell transcription factors MafA, Pdx1, and Neurod1 as well as other targets such as Glut2 and Gck.^[^ ^105^ ^]^

In addition to dedifferentiation, *β*‐cell senescence and aging also accelerate in type 2 diabetes. Senescence can be characterized with loss of *β*‐cell markers and detection of *β*‐galactosidase and p16^INK4A^ expression, which exacerbate the inflammatory state.^[^ ^106^ ^]^ This phenomenon is known as senescence associated secretory phenotype (SASP). When senescent cells secrete various modulators such as growth factors, cytokines and chemokines, SASP enforces cells to be in cell cycle arrest and activates immune response. SASP is mainly modulated by NF‐*κ*B, C/EBP and p53 transcription factors**.^[^** ^107^ **^]^** Moreover, removal of senescent cells (senolysis), by using transgenic INK‐ ATTAC mouse model or oral senolytic molecule (ABT263), decreases the rate of SASP and improves glucose handling and *β*‐cell function.^[^ ^108^ ^]^

Resident macrophages, located in mouse pancreatic islets, also play role in tissue homeostasis by promoting *β*‐cell mass and *β*‐cell function via various signaling molecules such as Smad7, vascular endothelial growth factor A (VegfA), and connective tissue growth factor (CTGF).^[^ ^109^ ^]^ In addition to macrophages, pericytes are also important players in pancreatic islets maintaining islet blood flow and regulating *β*‐cell function. Dysfunctions in pericytes lead to impaired *β*‐cell function and insulin secretion in diabetes.^[^ ^109^ ^]^ For instance, upon high glucose stimulation, pericytes secrete nerve growth factor (NGF), which binds to its receptor TrkA located on the *β*‐cells. Activation and phosphorylation of TrkA, in return, stimulates insulin secretion from the *β*‐cells. Disruption of NGF or TrkA impairs glucose handling and insulin secretion in mice.^[^ ^110^ ^]^ In addition to NGF/TrkA signaling axis, transcription factor 7 Like 2 (Tcf7l2) emerges as another factor that mediates pericyte dependent *β*‐cell regulation. Loss of Tcf7l2 in pancreatic pericytes impairs *β*‐cell function and exacerbates glucose intolerance in mice.^[^ ^111^ ^]^

Overall, loss of *β*‐cell mass and function is key to the development of full‐blown type 2 diabetes. Indeed, several of the type 2 diabetes treatments target *β*‐cells to induce insulin secretion such as sulfonylureas, dipeptidyl peptidase 4 inhibitors (DPP4i) as well as drugs that act as GLP‐1R and GPR40 agonists.^[^ ^112^ ^]^

## 6 Diabetic Complications

“Science, has been built upon many errors; but they are errors which it was good to fall into, for they led to the truth” said once the ingenious and talented French novelist Jules Verne, who himself developed type 2 diabetes in his fifties and unfortunately suffered miserably due to the diabetic complications in his late years.^[^ ^113^ ^]^

Diabetes is hardly a disease of mere elevation in blood glucose levels. In most cases, it brings along a plethora of complications in peripheral tissues such as kidneys, cardiovascular system, retina, the nervous system, and liver. Although the symptoms and the indications of these pathologies are quite well characterized, the underlying molecular mechanisms remain elusive. Hence, the existing therapies are not always as effective. The usual suspect leading to diabetic complications would be hyperglycemia; yet studies indicate that strict blood glucose control does not always prevent the progress of these pathologies let alone reversing it.^[^ ^114^, ^115^ ^]^ Large interventions trails such as UKPDS, VADT, ACCORD, and ADVANCED with glucose‐lowering approaches presented evidence for statistically significant reductions in relative risks for developing some of the diabetic complications, the rate of absolute risks however remained relatively small, such as a reduction of 0.28% for microvascular complications or 0.04% reduction in diabetic kidney disease in the UKPDS study.^[^ ^115^, ^116^ ^]^ Multifactorial interventions targeting hypertension, dyslipidemia, and microalbuminuria, along with hyperglycemia, on the other hand, were much more effective in reducing diabetic complications in Steno‐2 study.^[^ ^117^, ^118^ ^]^ In addition to strict glycemic control, insulin sensitizers were also associated with a reduction in diabetic complications but only with a 1.5% of absolute risk ratio for cardiovascular mortality and with a 1.8% absolute risk ratio for cardiovascular events in the case of pioglitazone.^[^ ^119^ ^]^ These data also raise the question how much hyperglycemia and insulin resistance play role in development of diabetic complications and whether they are just epiphenomena, that is, they might be symptoms of type 2 diabetes but might not necessarily contribute to its pathogenesis. In addition to hyperglycemia, deregulation of other cellular activities such as generation of the reactive metabolites may contribute to development of diabetic late complications.

According to Brownlee hypothesis a.k.a unifying hypothesis, hyperglycemia elevates ROS levels, which modify and impair the glycolytic enzyme glyceraldehyde 3‐phosphate dehydrogenase (GAPDH). Inhibition of glycolysis via inhibition of GAPDH diverts the upstream metabolites from glycolysis to glucose overutilization pathways, which are the following: 1) the polyol pathway; 2) the protein kinase C (PKC) pathway; 3) advanced glycation end product (AGE) formation pathway; and 4) the hexosamine pathway.^[^ ^120^ ^]^ These pathways lead to mitochondrial dysfunction and elevate ROS levels even further, contributing to disease progression and represent the root cause of diabetic complications.^[^ ^120^ ^]^ A major drawback of this hypothesis is the fact that ROS have a very short half‐life and spatially very limited actions. Although there are some studies that show patients with diabetes have elevated ROS, ROS levels do not necessarily change between patients with and without diabetic complications.^[^ ^121^ ^]^ Although mitochondrial impairment is relevant in terms of pathogenesis of diabetes and diabetic complications, the evidence for ROS‐induced mitochondrial dysfunction that leads to diabetic complications also remains elusive.

As both experimental and clinical approaches fail to provide solid and consistent evidence to support Brownlee hypothesis, researchers are investigating alternative pathways or metabolites that might play role in diabetic complications. Methylglyoxal (MG) represents one of these reactive metabolites; the levels of which increase upon hyperglycemic flux and impaired detoxification. One of the enzymes that play role in MG detoxification is Glyoxalase 1 (Glo1). Glo1 knockout flies have elevated levels of MG, which induces type 2 diabetes like phenotype such as insulin resistance, obesity, and hyperglycemia.^[^ ^122^ ^]^ Similarly, Glo1 knockout together with diet‐induced obesity elevates MG levels and induces type 2 diabetes like symptoms in zebrafish.^[^ ^123^ ^]^ In support of these findings, MG is also sufficient to induce retinopathy like lesions in rat models without inducing hyperglycemia,^[^ ^124^ ^]^ suggesting that accumulation of MG is creating a shortcut to develop diabetes‐like phenotype in the absence of hyperglycemia.

In addition to Glo1, MG can also be metabolized either by aldo–keto reductases (AKR) to hydroxyacetone or by aldehyde dehydrogenase (ALDH) to pyruvate. Compensatory MG detoxification by increased AKR and ALDH activities is more relevant in mammals, as unlike in *Drosophila* and zebrafish, loss of Glo1 do not elevate MG levels in mice.^[^ ^125^, ^126^ ^]^

### 6.1 Diabetic Kidney Disease

Diabetic kidney disease (DKD) (a.k.a diabetic nephropathy) develops as a microvascular complication of type 1 or type 2 diabetes with a prevalence rate of 30–40%. Diabetic kidney disease accounts for 30–47% of the end‐stage renal disease (ESRD) cases, being one of the major causes of diabetes related deaths. A better control of blood glucose levels correlates with a decrease in diabetic kidney disease progression. Yet, patients with diabetes still develop kidney disease despite tight control of blood glucose levels; suggesting additional insults such as oxidative stress and lipotoxicity might play a critical role as well.^[^ ^127^, ^128^ ^]^ Alternatively, hyperglycemic memory might explain why patients with strict blood glucose control still develop diabetic kidney disease.^[^ ^129^, ^130^ ^]^ The theory of metabolic memory initially emerged after large clinical trials that continued with a follow‐up period such as the DCCT trial with its follow‐up EDIC study for type 1 diabetes or the UKPDS trial for type 2 diabetes.^[^ ^131^, ^132^, ^133^, ^134^ ^]^ During the clinical trials, patients with diabetes received either standard or very intensive treatment. Once the trial ended, all patients switched to very intensive treatment and had similar HbA1c levels from then on. Nevertheless, the follow up studies showed that despite similar Hb1Ac levels, patients that had received standard treatment were at a higher risk of developing microvascular complications compared to patients that received intensive treatment before.^[^ ^131^, ^132^, ^133^, ^134^, ^135^ ^]^

The exact underlying mechanisms that lead to metabolic memory remain elusive. Nevertheless, the experimental studies in the laboratories show that irreversible genetic, epigenetic, cellular, and tissue‐level alterations that occur during episodes of hyperglycemia might lead to metabolic memory.^[^ ^136^, ^137^, ^138^, ^139^ ^]^

Kidney's function in filtration, ion homeostasis, and blood pressure rely heavily on its specialized anatomical structure and the multiple cell types it contains. Cell types that reside in kidney include podocytes, epithelial cells, and mesangial cells. Diabetic kidney disease involves both functional and morphological changes in the kidney tissue such as impaired podocyte function and its detachment from glomerular basement membrane (GBM). GBM itself also thickens due to ectopic accumulation of extracellular matrix components such as collagen type IV and VI as well as laminin and fibronectin. Together with mesangial matrix expansion, GBM thickening leads to glomerular sclerosis and tubule‐interstitial fibrosis, overall damaging kidney function, which presents itself as albuminuria and deteriorated glomerular filtration rate (GFR). Below, we will briefly describe the molecular mechanisms that underlie these morphological and functional changes in the kidney during diabetes.

**Figure 6:**
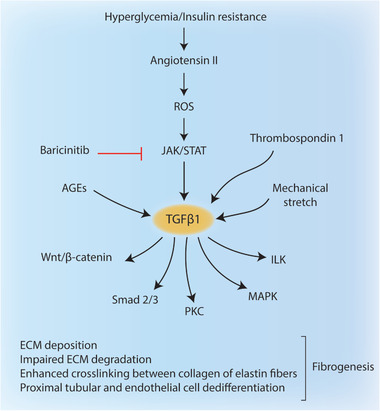
Diabetic kidney disease. Hyperglycemia and insulin resistance increase angiotensin II expression which activates TGF*β*1 via ROS and JAK/STAT signaling. Baricinitib, selective inhibitor of JAK1/2, can reduce albuminuria in type 2 diabetes patients. TGF*β*1 can also be activated via AGEs, mechanical stretch and thrombospondin 1. Activated TGF*β*1 stimulates a wide range of targets including Wnt/*β*‐catenin, Smad 2/3 complex, PKC, MAPK, and ILK to promote fibrogenesis in kidney.

Transforming growth factor‐beta 1 (TGF*β*1) plays a key role in development of fibrogenesis in the kidney by promoting extracellular matrix (ECM) deposition, impairing ECM degradation, enhancing crosslinking between collagen and elastin fibers, and activating proximal tubular and endothelial cell de‐differentiation (Figure 5).^[^ ^140^ ^]^ Hyperglycemia and insulin resistance increase the expression of Angiotensin II, which induces ROS production and activates TGF*β*1 signaling.^[^ ^141^, ^142^ ^]^ Aberrant Janus kinase–signal transducer and activator of transcription (JAK‐STAT) signaling also acts as an upstream regulator of TGF*β*1 signaling. Increased ROS levels due to hyperglycemia activate JAK2, which in turn increases the expression of TGF*β*1. Indeed, Baricinitib, a small molecule selective inhibitor of JAK1/2 effectively reduced albuminuria in type 2 diabetes patients in a phase 2 clinical trial study.^[^ ^143^, ^144^, ^145^ ^]^ Other stimuli that activate TGF*β*1 include mechanical stretch, AGEs and thrombospondin‐1. Smad2/3 complex, protein kinase C (PKC), p38 mitogen‐activated protein kinases (MAPK), interleukin like kinase (ILK) and Wnt/beta‐catenin signaling are among the downstream targets that mediate pro‐fibrogenic effects of TGF*β*1 (**Figure** **6**).^[^ ^146^, ^147^, ^148^, ^149^ ^]^ Although evidence suggest that TGF*β*1 has an established role in pathophysiology of diabetic kidney disease, therapies that target active TGF*β*1 unfortunately fail to show efficacy in clinical studies. Yet, targeting the latent form of TGF*β*1 instead of the active one holds promise for the treatment of diabetic kidney disease in the future.^[^ ^150^ ^]^

Aberrant lipid signaling is another emerging topic in the context of diabetic kidney disease Sphingomyelin phosphodiesterase acid‐like 3b (SMPDL3b) is a lipid draft enzyme, which is overexpressed in the kidneys of patients with type 2 diabetes.^[^ ^151^ ^]^ High SMPDL3b expression reduces Ceramide 1 phosphate (C1P) levels in the plasma membrane and leads to impaired insulin/Akt signaling in podocytes. Interestingly podocyte specific SMPDL3b deletion increases C1P levels and protects db/db mice from diabetic kidney disease. Administration of C1P exogenously, on the other hand, reduces albuminuria, blunts mesangial expansion and restores Akt signaling; overall ameliorating diabetic injury. These promising findings pave the way to the use of active lipids such as C1P for the treatment of diabetic kidney disease and potentially other diabetic complications.^[^ ^152^ ^]^

Excessive lipid accumulation in the kidney and the accompanying lipotoxicity are unfolding as relatively new concepts that play a role in development of diabetic kidney disease as well.^[^ ^153^ ^]^ Diabetic mice for instance overexpress junctional adhesion molecule‐like protein (JAML) in their podocytes that activates the Sirtuin‐1 (SIRT1) mediated Srebp1 signaling leading to excessive lipid accumulation and renal lipotoxicity. Podocyte specific deletion of JAML alleviates pathologies related to diabetic kidney disease such as lowering renal lipotoxicity impairing the progress of the disease.^[^ ^154^ ^]^

VEGF‐B also emerges as a critical target that elevates glomerular lipid content and causes insulin resistance in podocytes. Inhibition of VEGF‐B via pharmacological or genetic approaches ameliorates diabetic kidney disease in type 2 diabetes mouse models.^[^ ^128^ ^]^

The anti‐diabetic SGLT2 inhibitors (SGLT2i) not only prove to be effective in reducing blood glucose levels but they also show decent efficacy in slowing down the progression of diabetic kidney disease.^[^ ^155^, ^156^, ^157^, ^158^, ^159^, ^160^ ^]^ A very recent study by Maegawa and colleagues showed that increased ketone body production might be one of the mechanisms how SGLT2i have a protective role in diabetic kidney disease. Improved ketone body production in the kidney blunts hyperactivated mTORC1 signaling and attenuates renal damage.^[^ ^161^ ^]^ Enhanced mTORC1 signaling is a hallmark of diabetic kidney disease, which leads to podocyte and tubular damage by impairing autophagy, an essential cellular process for healthy podocyte function.^[^ ^162^, ^163^, ^164^ ^]^

In addition to increasing ketone body production, there are other potential mechanisms via which SGLT2i might have a renoprotective role. For instance, SGLT2 inhibitors initiate an anti‐inflammatory state in the body by reducing leptin, IL‐6, IL‐1*β* levels in the serum, while increasing adiponectin levels.^[^ ^165^, ^166^, ^167^ ^]^ In addition to these anti‐inflammatory benefits, SGLTi also alleviate the burden on the kidney through many different mechanisms including inhibition of oxidative stress, lowering of blood pressure, and delaying the progress of kidney fibrosis.^[^ ^168^, ^169^, ^170^, ^171^ ^]^

### 6.2 Cardiovascular Complications

Cardiovascular disease (CVD) is the most prevalent cause of mortality and morbidity among patients with diabetes. More than 30% of the type 2 diabetes patients suffer from cardiovascular complications and nearly half of type 2 diabetes related deaths occur due to CVD.^[^ ^172^ ^]^ CVD covers a plethora of dysfunctions in the cardiovascular system including atherosclerosis, myocardial infarction, heart failure, and cardiomyopathy. Although the number of studies that explore the diabetes and CVD connection are increasing exponentially, the exact pathogenic mechanisms remain elusive. In this section, we will summarize the newly identified signaling molecules that might play role in development of type two diabetes‐induced CVD.

**Figure 7:**
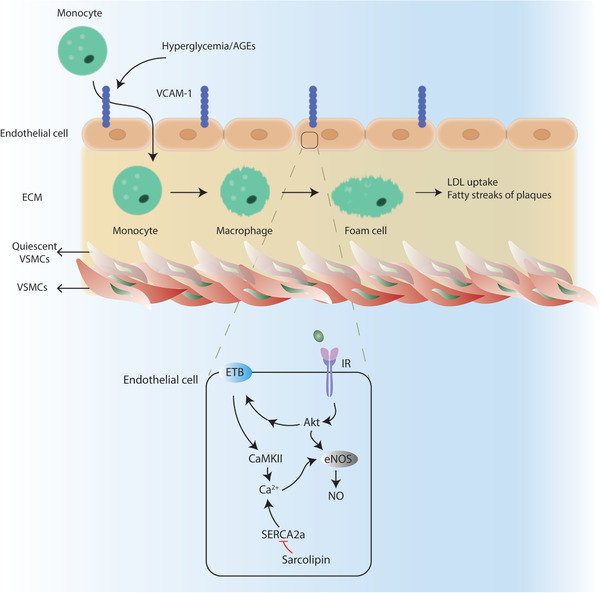
Cardiovascular complications. Hyperglycemia and AGEs cause endothelial cell dysfunction by increasing VCAM‐1 expression on the cell membrane. Monocytes bind to VCAM‐1 and infiltrate to ECM where monocytes differentiate into foam cells. Hyperglycemia also promotes quiescent vascular smooth muscle cell (qVSMC) activation which also contributes foam cell differentiation. In endothelial cells, eNOS can be regulated by Akt and CaMKII induced‐Ca^2+^ levels via endothelin B receptor (ETB). Sarcolipin inhibits SERCA2a function which exacerbates Ca^2+^ dysregulation.

Atherosclerosis the process of plaque formation inside the arteries and represents one of the most common form of CVD in patients with type 2 diabetes. Development of atherosclerosis is multifactorial and involves many different pathological stimuli and many different cell types. Hyperglycemia represents a great risk factor for atherosclerosis by promoting endothelial cell dysfunction, an early event during the development of atherosclerotic lesions. High blood glucose levels induce the production of AGEs that nonenzymatically attach to the proteins or lipids, altering their function. For instance, AGE‐modified proteins or lipoproteins bind and activate the receptor for AGEs (RAGE), which increases VCAM‐1 expression and enhances binding to monocytes that infiltrate into the ECM between the endothelial cells and smooth muscle cells (**Figure** **7**).^[^ ^173^, ^174^ ^]^

Hyperglycemia also activates the quiescent vascular smooth muscle cells (VSMC) that lie beneath the endothelial layer. When activated, VSMCs lose their contractility, gain proliferative, and migratory features along with enhanced inflammation and ECM production, altogether contributing to a proatherogenic phenotype.^[^ ^175^ ^]^ Activated VSMCs also contribute to the differentiation of monocytes into the foam cells, which involves extensive take up of the low‐density lipoproteins (LDL), leading to fatty streaks of the plaques at the artery walls (Figure 7).

Recently QKI‐7, an RNA binding protein, emerged as a key regulator of hyperglycemia‐induced vascular endothelial dysfunction. Patients with diabetes have increased QKI‐7 expression in their vessels. Interestingly, QKI‐7 binds and promotes mRNA degradation of its downstream targets CD144, Neuroligin 1 (NLGN1), and TNF‐*α*‐stimulated gene/protein 6 (TSG‐6), all of which are essential for EC function. Indeed in vivo knock down QKI7 restored endothelial cell function in mice, suggesting a potential role for QKI‐7 targeting in treatment of vascular complications of diabetes.^[^ ^176^ ^]^ Nitric oxide (NO) plays a protective role in development of atherosclerosis by regulating the contraction of vessels, inhibiting leukocyte attachment and platelet aggregation. NO was the first soluble gas to be identified as a signaling molecule. The enzyme responsible for intracellular NO production is called nitric oxide synthase (eNOS), which is regulated by signaling pathways such as PI3K/Akt, PKA, and Ca^2+^/calmodulin‐dependent protein kinase II (CaMKII). Although insulin resistance represents a great risk factor for development of atherosclerosis, the underlying mechanisms remain controversial and/or elusive. Akt can directly phosphorylate eNOS on S1177 and promote its function. Recent findings also show that PI3K/Akt signaling induces the expression of endothelin B receptor, which activates CamKII and elevates Ca^2+^ levels. Elevated Ca^2+^ level activates eNOS, which in turn increases NO levels, adding an extra protection against the proatherogenic insults (Figure 7).^[^ ^177^ ^]^

Dysregulated calcium signaling is a hallmark of diabetic hearts as hyperglycemia and AGEs disrupt the healthy interplay between the sarcoplasmic/endoplasmic reticulum Ca^2+^ATPase 2a (SERCA2a) mediated Ca^2+^ release and uptake by the sarcoplasmic reticulum. SERCA2a expression is indeed reduced in diabetic cardiomyocytes. Sarcolipin is one of the critical negative regulators of SERCA2a function. Increased sarcolipin expression in diabetic cardiomyocytes blunts the expression of DNA methyl transferase 1 (DNMT1) and DNMT3a, overall causing demethylation of its own promoter and increasing its own transcription. Elevated sarcolipin suppresses SERCA2a activity and exacerbates Ca^2+^ dysregulation leading to diabetic heart failure (Figure 7).^[^ ^178^ ^]^

Class II histone deacetylase (HDACs) are essential regulators of epigenetic changes upon stress signals. Interestingly, unlike the other members of HDAC family, HDAC4 can regulate *β*‐adrenergic signaling by responding to CaMKII and PKA signaling pathways. CaMKII phosphorylates HDAC4 at S467 and S632, and activates 14‐3‐3 mediated nuclear transport. PKA phosphorylates HDAC4 at S642 resulting in its proteolysis and cleavage of *N*‐terminal of HDAC4 (HDAC4‐NT). HDAC‐NT fragment protects from diabetic heart failure via hexosamine biosynthetic pathway (HBP) and *β*‐linked *N*‐acetylglucosamine O‐linked glycosylation (O‐GlcNAcylation) of calcium sensor STIM1.^[^ ^179^ ^]^

Exophers represent a very novel concept in the field of cellular waste disposal. Exophers are specialized structures that cells pack with protein aggregates and defective organelles such as mitochondria and exude them to extracellular milieu where they can be taken up by other cells. After only a couple of years of their original discovery in *Caenorhabditis elegans*, scientists discovered exophers in mice as well. Mouse cardiomyocytes employ exophers to maintain a healthy heart function. The cardiac muscle requires tremendous amount of energy made possible by mitochondria, which undergo a fast turnover due to their heavy use. Possibly, the cardiomyocytes speed up the mitochondria turnover, by simply packing the exophers with dysfunctional mitochondria and exude them into extracellular matrix where macrophages recognize them via their phagocytic receptor Mertk and engulf.^[^ ^180^ ^]^ Ablation of cardiac macrophages or Mertk deficiency leads to metabolic dysfunction in heart. Based on these exciting discoveries, it is very tempting to speculate that dysregulation of exopher‐mediated mitochondria disposal might play a role not only in cardiomyopathy but also in other diabetic complications as well.

### 6.3 Diabetic Retinopathy

Diabetic retinopathy is a common complication of diabetes. Almost 20% of the patients have diabetic retinopathy at the time of diagnosis with diabetes and overall 40–45% of the patients develop retinopathy during the course of the disease. Diabetic retinopathy involves dysfunction in two main cell types of the retina: endothelial cells of the retinal microvasculature and the pericytes that lie beneath the endothelial cells to support and regulate endothelial cell function. Briefly, hyperglycemia, oxidative stress and AGEs, impair the tight junctions between the endothelial cells and induce detachment and apoptosis of pericytes.

**Figure 8:**
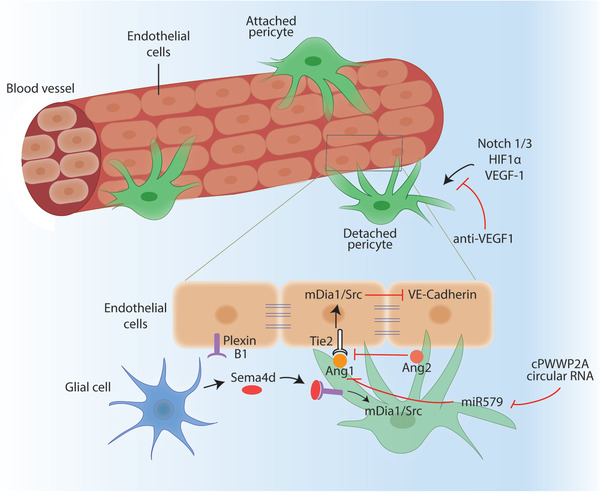
Diabetic retinopathy. Endothelial cells and pericytes are the two regulators of diabetic retinopathy. Hyperglycemia and oxidative stress cause pericyte detachment from the endothelial cells via Notch1/3, HIF1*α*, and VEGF‐1 signaling pathways. Anti‐VEGF‐1 therapies are used to inhibit detachment of pericytes. Glial cells express Sema4d during hypoxia and upon Sema4d binding to its receptor Plexin B1 in pericytes, mDia/Src pathway gets activated. Activated Src promotes VE‐cadherin internalization and loosens the tight junctions between endothelial cells. Ang1‐Tie2 binding also impairs Src function, while Ang2 inhibits Ang1‐Tie interaction. cPWWP2A circular RNA downregulates miR579, which in turn promotes Ang1 expression.

Pericyte loss is one of the very early pathologies in diabetic retinopathy, which renders these cells an important target for early interventions to prevent the further progress of the disease. Hyperglycemia leads to detachment of pericytes from the endothelial cells, which eventually leads to apoptosis and increases the blood‐retina barrier permeability. Signaling pathways that contribute to pericyte loss include Notch 1, Notch 3, hypoxia inducible factor 1 *α* (HIF1*α*), and VEGF‐1 (**Figure** **8**).^[^ ^181^, ^182^ ^]^

Hyperactive VEGF‐1 signaling contributes significantly to the progression of diabetic retinopathy by inducing highly unstructured, disorganized neovascularization of endothelial cells. Anti‐VEGF‐1 therapies have been very effective to delay the disease progression. Yet, not all patients respond to VEGF‐1 treatment equally. Recent findings indicate that Semaphorin 4d (Sema4d) levels in the body fluids can successfully predict whether patients will respond to anti‐VEGF1 therapy or not.^[^ ^183^ ^]^ Non‐ or little responders of the anti‐VEGF‐1 therapy have elevated levels of Sema4d in the aqueous fluid of the eye. Sema4d not only acts as a biomarker but also plays a significant role in progression of diabetic retinopathy. Indeed, a combination therapy of anti‐VEGF1 and anti‐Sema4d might be a better alternative compared to only anti‐VEGF1 treatments. Sema4d is a membrane bound protein whose expression elevated upon hypoxia in the retinal glial cells. Once shedded at the cell membrane by ADAM17, Sema4d binds to its receptor PlexinB1 at the surface of pericytes and endothelial cells, which activates downstream signaling of mDia1/Src pathway. Activation of Src contributes to phosphorylation and internalization of vascular endothelial cadherin (VE‐Cadherin) which loosens the tight junctions and contributes to vascular leakage exacerbating the diabetic retinopathy (Figure 8).^[^ ^183^, ^184^ ^]^ Hence, Sema4d sets a nice example of how the crosstalk between retinal glial cells and the pericytes holds critical function to maintain a healthy vasculature in the eye.

Other upstream regulators of Src include Angiopoietin 1 (Ang1), which is expressed and secreted by pericytes and binds to its receptor Tie2 on endothelial cells. Activated Ang1/Tie2 signaling promotes TGF*β* and platelet‐derived growth factor (PDGF) signaling in endothelial cells, which stabilize the intercellular interactions. Ang1/Tie2 signaling also impairs Src function to promote the tightening of cell–cell junctions between epithelial cells blunting vascular hyperpermeability.^[^ ^184^ ^]^ Ang2, on the other hand, acts as an antagonist to blunt the Ang1/Tie2 signaling and promote blood retina barrier permeability (Figure 8).^[^ ^185^ ^]^

Similar to glial cell–pericyte crosstalk, pericyte–endothelial cell crosstalk is also essential for a healthy retinal vasculature. One of the newly identified mediators of pericyte–endothelial cell crosstalk is the circular RNA called cPWWP2A. Circular RNAs are a group of non‐coding RNAs with a closed loop structure and usually act as sponges to downregulate the action of their target microRNAs. High glucose levels upregulate the expression of cPWWP2A in pericytes, which acts as a sponge to impair miR‐579 function and upregulate Ang1/Occludin/SIRT1 expression. cPWWP2A is also packed in exosomes and secreted into the pericyte medium to regulate the proliferation, migration, and tube formation of retinal endothelial cells.^[^ ^186^ ^]^ Similar to cPWWP2A, cZNF532 is another novel circular RNA that plays role in controlling pericyte function and vascularization. cZNF532 acts as a sponge to downregulate miR‐29a‐3p, which in turn increases the expression of miR‐29a‐3p targets neuron‐glial antigen 2 (NG2), lysyl oxidase like 2 (LOXL2), and cyclin‐dependent kinase 2 (CDK2). Downregulation of cZNF532 impairs NG2, LOXL2, and CDK2 expression, which contribute to pericyte degeneration and vascular dysfunction.^[^ ^187^ ^]^

Chronic inflammation due to elevated levels of oxidized lipoproteins, free radicals and AGEs is also a hallmark of diabetic retinopathy. Proinflammatory cytokines that contribute to pathogenesis of diabetic retinopathy such as vascular leakage, endothelial cell apoptosis and capillary degeneration including IL‐6, IL‐1*β*, IL‐17A, MCP‐1, and TNF*α*. Recent findings show that the prostaglandin E2 and its cognate EP2 receptor plays role in inducing the expression of not only IL1*β* but also the inflammasome NLR family pyrin domain containing 3 (NLRP3) signaling in diabetic retinopathy.^[^ ^188^, ^189^, ^190^ ^]^

Lipids are also emerging as secondary messengers that play role in progression of diabetic retinopathy. Ceramide 6, for instance, induces the expression of regulated in development and DNA damage responses 1 (REDD1), which impairs JNK function and prevents apoptosis.^[^ ^191^ ^]^ Other lipids that might have a protective role in pathogenesis of diabetic retinopathy include decosahexaenoic acid (DHA) and eicosapentanoic acid (EPA); whereas 12‐hydroxyeicosatrienoic acids (12‐HETE) or 15S‐HETE seem to exacerbate the progression of diabetic eye disease.^[^ ^192^ ^]^

Inhibitors of dipeptidyl peptidase 4 (DPP4i) are commonly used to treat type 2 diabetes. Linagliptin, sitagliptin, and diprotin A are the main DPP4i used against diabetic retinopathy. DPP4ii are regulated upon glucagon like peptide (GLP) 1 binding to GLP 1 receptor (GLP 1R) as well as other substance specific interactions such as high mobility group box 1 (HMGB 1) and stromal cell derived factor 1*α* (SDF 1 *α*). SDF 1*α* and VEGF work synergistically on neovascularization. In the oxygen induced retinopathy (OIR) model, a retinal neovascularization model, diprotin A induced aggravated permeability and promoted proangiogenic response leading to revascularization of avascular zone in retina. Interestingly, linagliptin acts more specific towards DPP 4 rather than other DPP family members and linagliptin treatments showed GLP1R independent anti angiogenic effects mediated by an inhibition of VEGFR signaling.^[^ ^193^ ^]^

### 6.4 Diabetic Neuropathy

Nearly half of diabetes patients experience complications in their autonomic and peripheral nervous system, known as diabetic neuropathy. In most cases, diabetic neuropathy affects the peripheral sensory nerve endings in hands and lower limbs causing pain, burning, tingling feeling as well as numbness. As the disease progresses, motor nerve endings at lower extremities get damaged, causing loss of balance and numb foot with loss of sensation. In addition to peripheral damage in the nerves, there are also cases where diabetic neuropathy develops at the proximal regions such as the thigh or pelvic and presents a proximal‐to‐distal gradient.^[^ ^194^, ^195^, ^196^ ^]^

**Figure 9:**
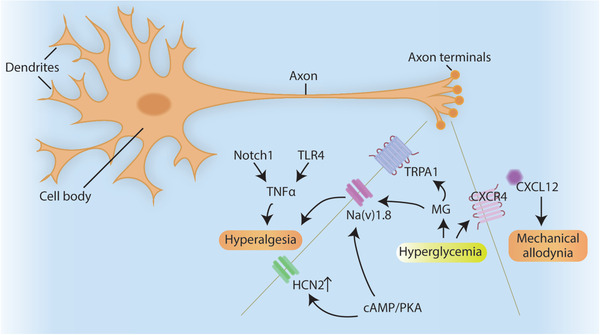
Diabetic neuropathy in axon terminals. Notch and TLR4 promotes the expression of TNF*α* which exacerbates hyperalgesia. Increased cAMP/PKA signaling leads to aberrant Na(v)1.8 channel and HCN2 channel function which also leads to hyperalgesia in diabetes. Hyperglycemia induced Methylglyoxal (MG) also modifies Na(v)1.8 and TRPA1 receptors and promotes hyperalgesia. CXCL12 and CXCR4 are novel targets that can initiate mechanical allodynia in diabetic neuropathy.

Diabetes‐induced activation of polyol pathway and concurrent depletion of NADPH and glutathione (GSH) lead to accumulation of MG and AGEs which impair nerve function. One of the targets of MG include the voltage‐gated sodium channel Na(v)1.8 which leads to abnormally increased sensitivity to pain a.k.a hyperalgesia in diabetes.^[^ ^197^ ^]^ Other upstream regulators of Na(v)1.8 include cAMP and PKA. cAMP also elevates the levels of hyperpolarization‐activated cyclic nucleotide–gated 2 (HCN2) ion channels in nociceptive nerve fibers. Hyperactivated HCN2 in Na(v)1.8 positive neurons drive pain in mouse models of diabetic neuropathy and inhibition of HCN2 alleviates pain in both type 1 and type 2 diabetes mouse models (**Figure** **9**).^[^ ^198^ ^]^ In addition to hyperalgesia, diabetic neuropathy also involves mechanical allodynia and small fiber degeneration. Mechanical allodynia is a common phenomenon in diabetic neuropathy, which means induction of pain due to stimuli that under normal conditions do not provoke pain. Recent findings indicate the C‐X‐C motif chemokine 12 (CXCL12)/ C‐X‐C chemokine receptor type 4 (CXCR4) signaling axis might play a critical role in initiation of mechanical allodynia in diabetic neuropathy (Figure 9).^[^ ^199^ ^]^

MG modification of ligand‐gated ion channel transient receptor potential cation channel, subfamily A, member 1 (TRPA1) also increases pain related hypersensitivity in diabetic neuropathy (Figure 9).^[^ ^200^ ^]^ The neuronal oxidative/nitrosative stress also activates MAPK, JNK and nuclear factor “kappa‐light‐chain‐enhancer” of activated B‐cells (NFĸB) pathways that further promote cytokine production and inflammation contributing to diabetic neuropathy. Recent advances in high throughput analysis such as microarrays and RNA‐Seq indicated that pathways that regulate inflammation and lipid metabolism might play a critical role in development of diabetic neuropathy. Potential targets of such analyses include PPAR*γ*, Apoliprotein E (ApoE), and leptin.^[^ ^201^ ^]^ Genetic risk factors are also a component of diabetic neuropathy. Specific polymorphisms in proinflammatory and lipogenic genes such as APOE, SREBP‐1, NF‐ĸB, nitricoxidesynthase 3 (NOS3), Toll‐like receptor 2 (TLR2) and TLR4, are associated with type 2 diabetes and diabetic neuropathy.^[^ ^202^, ^203^ ^]^ When activated TLR4 initiates a cascade of signaling events that promote the expression and secretion of TNF*α* creating a neuroinflammatory state. Inhibition of Notch1 or TLR4 reduces TNF*α* levels and alleviates mechanical allodynia while improving thermal hyperalgesia thresholds.^[^ ^204^ ^]^

Emerging evidence suggests lack of insulin and insulin resistance in sensory nerves might also play a role in development of diabetic neuropathy. Insulin acts as a neurotrophic hormone required to maintain normal nerve functions. Lack of insulin signaling in diabetes leads to mitochondrial dysfunction, impaired neurochemical synthesis and reduced regenerative capacity, all of which might contribute to development of diabetic neuropathy.^[^ ^205^ ^]^

A major component of the peripheral nervous system is the Schwann cells that surround the sensory axons for protection and survival. Hyperglycemia in diabetes not only impairs the sensory neurons but also the Schwann cell function which leads to myelin disruption, impaired axon conduction, and compromised regeneration in diabetic neuropathy via deregulation of different targets such as MAPK, p75 neurotrophin receptor (NTR), and *β*‐nerve growth factor (NGF) as well as neurotrophic factor‐3 (NT‐3).^[^ ^206^ ^]^

### 6.5 Liver Fibrosis

Non‐alcoholic steatohepatitis (NASH) and liver fibrosis are also emerging as late complications of diabetes. Under normal conditions, liver is great at handling acute stress conditions and regenerate when required. Apoptosis of damaged cells is a crucial part of this regeneration process, which needs to be under control for healthy liver function. Constant exposure to hyperglycemia, insulin resistance, and excessive lipid accumulation, on the other hand, induce a chronic inflammatory state where lipotoxicity and oxidative stress contribute to development of NASH from a relatively benign state of non‐alcoholic fatty liver disease (NAFLD). Unlike NAFLD, NASH is hardly reversible which progresses further into fibrosis and eventually cirrhosis, when not managed properly. Currently there are no FDA approved therapies to treat NASH and/or liver fibrosis, which represent the most common cause of liver transplants worldwide.

Mitochondrial non‐coding RNAs are recently identified as contributing factors to NASH development. Steatohepatitis‐associated circRNA ATP5B regulator (SCAR), which is located in mitochondria, inhibits mitochondrial ROS output and fibroblast activation.^[^ ^207^ ^]^

AMPK; a central regulator of cell metabolism, also plays a crucial role in maintaining hepatic homeostasis. NASH development suppresses the function of AMPK, which under normal conditions phosphorylates and inhibits caspase 6 activity. Downregulation of AMPK during NASH leads to hyperactivation of Casp 6 causing excessive apoptosis in the liver tissue which exacerbates inflammation and liver injury.^[^ ^208^ ^]^ One of the key mediators of liver inflammation and injury is JNK1. JNK1 induces the expression of pro‐inflammatory cytokines and chemokines such as IL‐6, MCP‐1 via its targets c‐jun and c‐fos. Apoptosis signal‐regulating kinase 1 (ASK1) represents one of the critical upstream activators JNK1. Ask1 homodimerization is indispensable for its activity, which is impaired by direct binding of Casp 8 and FADD like apoptosis regulator (CFLAR) protein. Interestingly hepatic CFLAR expression is reduced during metabolic syndrome and NASH. Adeno associated virus (AAV) mediated hepatic reconstitution of CFLAR improves glucose tolerance and alleviates liver fibrosis both in mice and monkeys, rendering it an attractive target for development of novel therapies against NASH.^[^ ^209^ ^]^

In addition to hepatocytes, hepatic stellate cells also play an important role in development of NASH and liver fibrosis. When stimulated by inflammatory cytokines or growth factors such as TGF*β*1, stellate cells get activated and undergo epithelial to mesenchymal transition, gain fibroblastic features and start to proliferate. Upon activation, stellate cells also increase the production of extracellular matrix components such as collagen contributing to liver stiffness and fibrosis.

The crosstalk between hepatocytes and stellate cells exhibit a major factor that accelerates fibrotic process. TAZ protein, for instance, initiates a signaling cascade to activate the expression of secretory factor Indian Hedgehog (Ihh). Ihh secreted from hepatocytes binds to smoothened receptor on stellate cells and induces the expression of pro‐fibrogenic genes and promotes proliferation. TAZ silencing in hepatocytes delays the progression of NASH and alleviates inflammation.^[^ ^210^ ^]^

### 6.6 Other Complications of Type 2 Diabetes

In addition to relatively well‐characterized pathologies explained above, emerging data indicate restrictive lung diseases such as lung fibrosis might be a late complication of diabetes as well.^[^ ^211^, ^212^ ^]^ Since it is a relatively new concept, the number of studies that unravel the potential mechanisms are limited. Nevertheless, compelling evidence suggests that hyperglycemia/oxidative stress‐induced DNA damage might play a role in diabetes‐associated lung fibrosis. RAGE plays role in DNA damage repair pathway and AAV‐mediated delivery of hyperactive phospho‐mimetic RAGE reverses diabetes‐associated fibrosis both in the lungs and kidneys of mice with diabetes.^[^ ^213^, ^214^ ^]^

Between 13%–24% of patients with diabetes present cognitive dysfunction in many multiple forms such as dementia, impaired attention, poor verbal memory, and deficits in executive functioning. The insulin resistance in the brain might be one of the mechanisms that lead to impaired neural function. Other potential causes include neuroinflammation, deregulated iron metabolism, and accumulation of hyperphosphorylated tau protein, which creates protein aggregates. Indeed, there is a strong association between type 2 diabetes and Alzheimer's disease.^[^ ^215^ ^]^

## 7 Conclusion

Although diabetes mellitus is one of the earliest described diseases of the human history, there is still no cure for it. Currently the existing therapies for type 2 diabetes target reducing blood glucose levels and alleviating the symptoms of accompanying complications. Although there are cases where bariatric surgeries, intermittent fasting, or certain diets such as ketogenic diet improve type 2 diabetes; these interventions are either highly invasive or the diet regimens are hard to follow up in the long run, respectively.

Thus, main research efforts should aim for the development of novel preventive and therapeutic concepts. This will likely include the more thorough investigation of SGLT2 inhibitors and GLP1 receptor agonists, which show effective clinical outcomes not only in reducing blood glucose levels, but also in alleviating the diabetic complications particularly in cardiovascular system and kidney.^[^ ^216^, ^217^ ^]^ Furthermore, the clinical validation of uni‐molecular, dual agonists, islet cell replacement as well as novel RNA‐based therapies for tailored diabetes treatment will certainly contribute to more efficacious therapies in type 2 diabetes and related complications.

Emerging insights into mechanisms of diabetic long‐term complications that go beyond the simple glucose‐centric view and incorporate as‐yet unexplored organ complications will be the basis for intensified research efforts to prevent or even reverse long‐term complications.

In addition to a better understanding of mechanisms playing role in pathogenesis of type 2 diabetes, patient stratifications based on these very pathogenic mechanisms will pave the way to more effective treatments for type 2 diabetes and its complications.

Major progress in these areas will eventually move us closer to our vision to make type 2 diabetes a livable and most importantly a curable disease in the future.

## Conflict of Interest

The authors declare no conflict of interest.
